# Seed Germination in Oil Palm (*Elaeis guineensis* Jacq.): A Review of Metabolic Pathways and Control Mechanisms

**DOI:** 10.3390/ijms21124227

**Published:** 2020-06-13

**Authors:** Jing Cui, Emmanuelle Lamade, Guillaume Tcherkez

**Affiliations:** 1 Research School of Biology, ANU Joint College of Science, Australian National University, Canberra, 2601 ACT, Australia; jing.cui@anu.edu.au; 2 CIRAD, UPR Systèmes de Pérennes, 34398 Montpellier, France; emmanuelle.lamade@cirad.fr; 3 Systèmes de Pérennes, Université de Montpellier, CIRAD, 34398 Montpellier, France; 4 Institut de Recherche en Horticulture et Semences (IRHS), Université d’Angers, INRAE Angers, 42 rue Georges Morel, 49782 Beaucouzé cedex, France

**Keywords:** oil palm, seed, lipid remobilization, germination, metabolism, haustorium

## Abstract

Oil palm is an oil-producing crop of major importance at the global scale. Oil palm mesocarp lipids are used for myriads industrial applications, and market demand has been growing for decades. In addition, oil palm seeds are oleaginous, and the oil extracted therefrom can be used for several purposes, from food to cosmetics. As such, there is a huge need in oil palm seeds to maintain the global cohort of more than 2 billion trees. However, oil palm seed germination is a rather difficult process, not only to break dormancy, but also because it is long and often reaches lower-than-expected germination rates. Surprisingly, despite the crucial importance of germination for oil palm plantation management, our knowledge is still rather limited, in particular about germinating oil palm seed metabolism. The present review incorporates different pieces of information that have been obtained in the past few years, in oil palm and in other palm species, in order to provide an overview of germination metabolism and its control. Further insights can also be gained from other oleaginous model plants, such as *Arabidopsis* or canola, however, palm seeds have peculiarities that must be accounted for, to gain a better understanding of germinating seed metabolism.

## 1. Introduction

**Figure 1:**
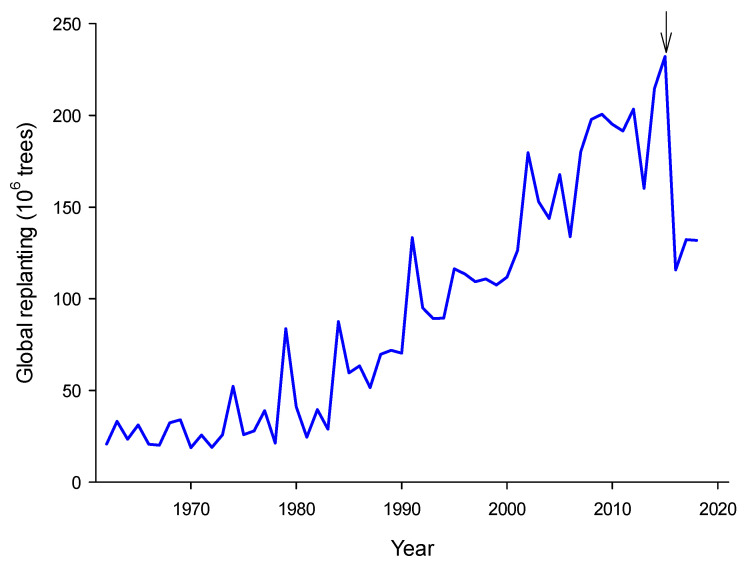
Estimated global demand in oil palm seedlings for new plantations and turn-over of old plantations. Computed from total world oil palm cultivation surface area using an average density of 143 trees ha^−1^ (FAO 2018). The arrow shows the year 2015 where total cultivated area reached a maximum.

Oil palm (*Elaeis guineensis* Jacq.) is presently the most productive oil crop, with a total global cultivation area of 19 million ha, and a recorded production of 0.27 gigatons of fruits and 71.4 megatons of palm oil [1]. This huge cultivated surface area represents about 2.7 billion trees. On average, oil palm fields are generally renewed every 25 y, thus having an average replanting turn-over of 4% y^−1^ [2], meaning that each year, about 100 million trees have to be replaced. In addition to the increase in cultivated surface area in the past decades, this generates a huge demand in oil palm sapling cultivation in nurseries and, of course, in seedling production from seed germination or in vitro cloning (Figure 1) (for a review on oil palm seed distribution and management, see [3]). Developing new crosses or hybrids (e.g., *E. guineensis x oleifera*) associated with better resistance to diseases (such as fatal yellowing) also requires growing palms from seeds. However, seed germination is still a hurdle in oil palm cultivation—as in many palm species—because of the relatively short seed storage time (preferably less than 16 weeks), methods required to break dormancy, and long germination and seedling establishment time, up to 9 months in nursery and up to several years in nature.

**Figure 2:**
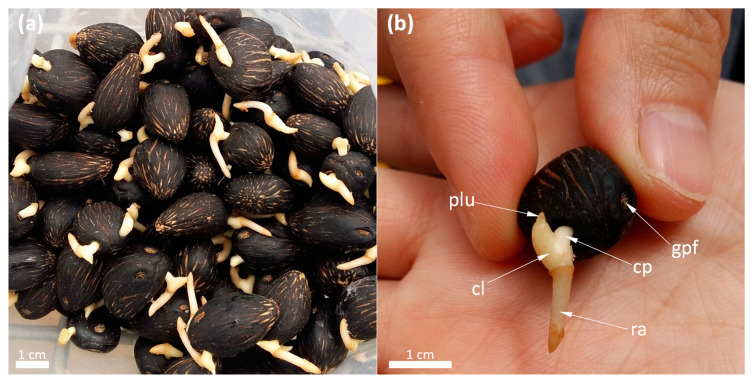
Photographs of germinated oil palm seeds: overview of seeds as they are in nurseries upon delivery, in polyethylene bag (**a**); anatomy of a germinated seed (**b**), showing one the germinative pore (gpf), the cotyledonary petiole (cp), cotyledonary ligule (cl), the radicle (ra) and the plumule (plu). Photographed seeds are at stage IV (see Figure 3 for the definition of stages). Photographs: © the author (Jing Cui).

Surprisingly, despite these difficulties to germinate and the high demand at the global scale, the physiology of oil palm germination and seedling establishment is not very well known. Most metabolic studies were carried out in the 1980s [4,5,6,7] and, since then, there has been very little progress in our knowledge of molecular actors and metabolic regulations involved in germination stricto sensu, seed storage remobilization and seedling development of oil palm. This species produces oily (oleaginous) seeds encapsulated into a hard endocarp layer, forming a kernel (photographs in Figure 2). As such, oil palm seeds are also used as a source of oil (palmist oil), with industrial applications that differ from mesocarp oil. The prevalence of lipids in kernels also implies specific metabolic pathways of remobilization, that is, lipid degradation and conversion to sugars. In this brief review, we will summarize metabolic aspects of oil palm seed germination (lato sensu) using recent data obtained not only in oil palm but also in other closely related palm species of the same botanical tribe (*Cocoeae*). The importance of primary metabolism in defining seed quality has been summarized recently [8]. We thus take advantage of the present review to emphasize metabolic aspects that are currently unknown and deserve further research, in particular, to find new avenues to optimize oil palm seed germination.

## 2. Setting the Scene: Specific Germination Stages

**Figure 3:**
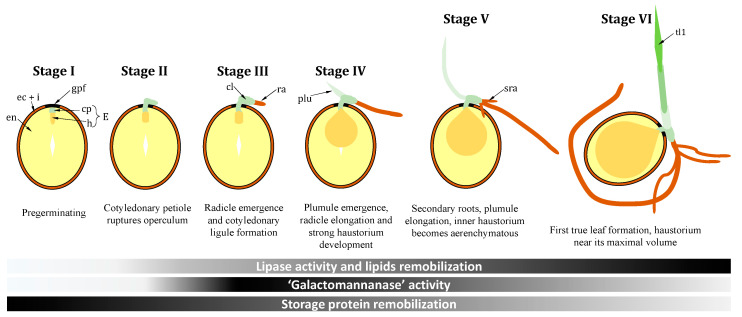
Simplified overview of oil palm seed germination. Germination is spelt out into six steps, following palm seeds germination description in stages I to VI proposed by Mazzotini-dos-Santos et al. (2016). The overall seed structure is simplified here and only shows endocarp and integuments (ec + i), endosperm (en), the germinative fibrous pore (or operculum; gpf) and the embryo made of the haustorium primordium (h) and the cotyledonary petiole (cp). The haustorium and the cotyledonary petiole form the embryo (E). Once the cotyledonary petiole pierces the operculum, the radicles (ra) emerges and a cotyledonary ligule forms (cl). Then, the shoot primordium (plumule; plu) emerges, secondary roots develop (sra) and eventually, the shoot produces successive leaves including the first true leaf forming a photosynthetic blade (tl1). This figure uses the term “galactomannanase” to encapsulate all enzymatic activities (such as β-endomannanases) involved in galactomannanes remobilization.

By definition, germination is the process by which seeds break their quiescent life, imbibe and start development so that the radicle pierces seed envelopes (integuments and endocarp). In palm tree species, such as oil palm, this definition has to be modified, to take into account the fact that the first anatomical structure that pierces seed envelopes is not the radicle, but a specific structure that represents the cotyledonary petiole. In other words, radicle development (as well as plumule development) are part of post-germinative events. The cotyledonary petiole is associated with (and attached to) the cotyledon (equivalent to the scutellum in grasses), which here forms a haustorium (Figure 2; Figure 3). The haustorium has a critical metabolic role since it interfaces the endosperm, which contains reserves that are digested and remobilized to sustain seedling development. That is, the haustorium plays multiple roles [4,9,10]: (*i*) induces enzymatic activities in the endosperm to allow remobilization; (*ii*) synthesizes digestion enzymes; (*iii*) transfers metabolites from the digestion zone to the seedling; (*iv*) involves specific metabolic pathways to coordinate post-germination events. The haustorium increases in size up to c. 90 days and its metabolic degradation starts at about 160 days [11]. Histological and metabolic steps in germination stricto sensu and post-germination have been recently described precisely in macaw palm (*Acrocomia aculeata*) [9], which may serve as a model species for palm seed germination. There, seven developmental stages have been recognized (described in Figure 3). Germination stricto sensu represents stages I-II, while stages III-VI are post-germinative. Importantly, these stages are associated with differences in prevalent metabolic activities. In fact, lipids are not the first seed reserves to be remobilized. Endosperm proteins appear to be broken down first, rapidly followed by carbohydrates [9,12]. Interestingly, reserves remobilization seems to start very early in palm seeds (at stage I) and, thus, is part of germination stricto sensu, whereas in most species (such as *Arabidopsis* or canola, two other oleaginous seeds), it starts in stage III, and is thus a post-germinative phenomenon.

## 3. Non-lipid Reserve Remobilization

**Figure 4:**
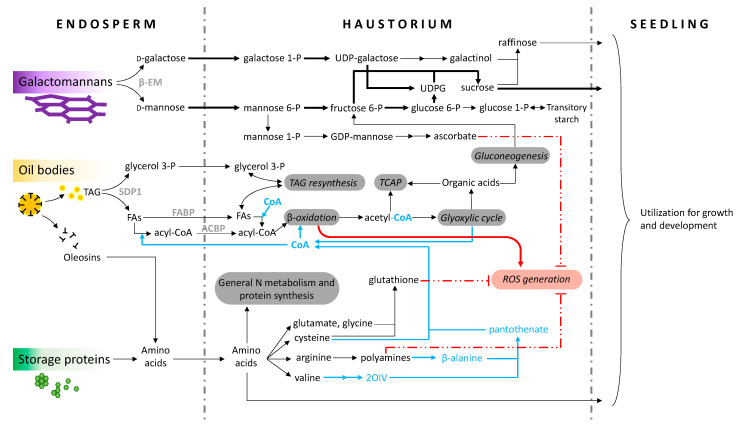
Overview of metabolic pathways involved in oil palm seed germination showing the role of the haustorium. For clarity, this figure does not show all intermediates, transporters and possible metabolic routes, and does not recall time windows for reserve utilization (shown in Figure 3). Enzymes discussed in text are in bold grey. Reactive oxygen species (ROS) generation and mitigation is shown in red. Coenzyme A cycle and synthesis are shown in light blue. Sucrose synthesis from galactomannanes degradation is shown in thick black arrows. Abbreviations: 2OIV, 2-oxoisovalerate; ACBP, acyl-CoA binding proteins; βEM, β-endomannanases; FABP, fatty acid binding proteins; FAs, fatty acids; ROS, Reactive oxygen species; TAG, triacylglycerols; TCAP, tricarboxylic acid pathway; UDPG, UDP-glucose. Here, oil bodies are shown as comprising oleosins (see further discussion in main text).

Non-germinated oil palm seeds contain (in % dry weight) *c*. 53% lipids, 38% insoluble carbohydrates and fibers, 8% proteins, 3% inorganic salts and 1% starch and soluble sugars [11,13,14]. Reserve proteins (which have been better characterized in embryos than in endosperm) are mostly made of globulins and albumins [15,16,17,18,19]. It is believed that the endosperm itself produces proteases to catalyze reserve protein hydrolysis, which are activated by a yet unknown signal coming from the haustorium [9]. It is possible that a hormone (such as a gibberellin) is involved but this is presently uncertain (see below the section Control of Germination). In other species with oleaginous seeds, such as *Arabidopsis*, proteins can also be directed to hydrolysis via the proteasome (summarized in [20]). Presumably, amino acids produced therefrom can easily penetrate into the haustorium via transporters (currently not characterized in oil palm). It is worth noting that reserve proteins in oil palm seeds have a high proportion of N-rich amino acids such as glutamine (20–30%) but quite interestingly, also arginine (10–20%) [16]. Arginine is not only a source of nitrogen (arginine cleavage by arginase yields urea which can be broken down to ammonium by urease, and ammonium can in turn be refixed by glutamine synthetase), but also a precursor of polyamines (putrescine, spermidine, spermine) and thus β-alanine (Figure 4).

Oil palm seed reserve carbohydrates are mostly in the form of insoluble (galacto) mannanes, which have been shown by ^13^C-NMR spectroscopy to be mostly made of linear mannans with a small proportion (less than 20%) of galactomannans [21]. These polymers are accumulated in cell walls and thus their degradation may participate in endosperm cellular thinning to favor cotyledonary petiole emergence, as suggested in macaw palm [10]. Galactomannans hydrolysis (via β-endomannanase activity) has been shown in situ by histochemistry endosperm, but not in the haustorium in macaw palm [9], suggesting that β-endomannanases are neither produced nor secreted by the haustorium epidermis, but are synthesized by the endosperm itself. Cell wall carbohydrate degradation probably also involves—like in other crops [22]—some other enzymatic activities, such as galacturonases, glucanases, cellulases, expansins, etc., in particular to facilitate the cotyledonary petiole emergence. In fact, in palm species other than oil palm, the degradation of pectins in the micropylar region (beneath the germination pore, Figure 3) has been demonstrated [10].

Although this has not been shown directly (typically using ^13^C or ^14^C isotope tracing), it is likely that sugar residues produced by (galacto)mannans hydrolysis (mannose, galactose) are not consumed by haustorium catabolism to a high extent, since β-endomannanase hydrolytic activity peaks at stage III [9], while lipid remobilization has already started. That is, haustorium catabolism mostly utilizes products of lipid degradation. At this stage, it is worth mentioning that in principle, mannose 6-phosphate may be used to synthesize ascorbate via GDP-mannose [23], and galactose may not only form UDP-glucose (thereby sustaining sucrose synthesis), but also galactinol for raffinose synthesis, which is also involved in sugar transport (Figure 4). Galactinol and raffinose have indeed been shown to be part of oil palm metabolome in all organs [24].

## 4. Lipid Remobilization

Kernel lipids are mostly made of triacylglycerols (TAG), while other compounds are minor constituents (such as α-tocopherol, phospholipids or carotenoids). Kernel oil (palmist oil) is richer in saturated fatty acids than mesocarp oil (about 89% fatty acids are saturated); also, medium chain fatty acids prevail, in particular lauric acid (C12:0) and myristic acid (C14:0), which account for up to 55 and 18% of total kernel fatty acids [25]. This seed lipid composition has been shown to be representative of many tropical palms growing in areas where the average temperature is high enough to avoid undesirable phase transition to solid fat [26]. Oil palm mesocarp oil bodies are believed to be devoid of oleosins [27], however, the oil palm genome contains nine oleosin-like proteins, suggesting that unlike mesocarp lipids, kernel oil bodies might comprise oleosins. Future proteomics analyses of pure kernel oil bodies are warranted to provide further insight on this aspect.

In macaw palm, both the endosperm and the haustorium synthesize a lipase that cleaves esterified fatty acids of oil bodies [9]. Surprisingly, enzymatic assays carried out with oil palm endosperm and haustorium samples have been unsuccessful to show lipase activity, in contrast to seedling tissues [7]. As shown in other species, such as *Arabidopsis* [28,29] the lipase that cleaves TAG is certainly of the Sugar Dependent 1 (SDP1) family, and in fact, the oil palm genome encodes for two SDP1-like lipases (scaffolds p5.00-sc00032-p0085 and p5.00-sc00086-p0021 in [30]). The failure to retrieve lipase activity in haustorium and endosperm samples could have come from the fact that a physical association with proteins and, perhaps, peroxisomes is necessary [29]. Alternatively, lipid remobilization could also involve autophagy, as recently suggested for other seed systems [20]. In any case, free fatty acids liberated by TAG hydrolysis must then be transferred to the haustorium, where enzymes of β-oxidation are located [5,7]. In other words, fatty acids generated by lipid cleavage must go through several membranes (two plasma membranes (endosperm and haustorium cells) and then the peroxisome membrane). This transport is certainly in the form of coenzyme A (CoA) esters produced by both endosperm and haustorium fatty acid-coenzyme A synthetase [5]. Transport into the peroxisome certainly involves an ABC family transporter homologous to the ABC transporter COMATOSE (CTS; oil palm has five CTS with a protein sequence identity of *c*. 75% compared to *Arabidopsis* CTS) [31], as well as acyl-CoA binding proteins (ACBP) and fatty acid binding proteins (FABP) [32]. Both ACBP and FABP are represented in oil palm genome (where there is actually only one annotated FABP, encoded by p5.00-sc00100-p0042, and six ACBP). Lipid quantification and tracing experiments with ^14^C-labelled laurin (trilauroylglycerol) or free fatty acids have clearly established that the haustorium does not only accumulate free fatty acids, but also resynthesizes lipids from imported fatty acids, such as TAG, diacylglycerols and monoacylglycerols, but also polar lipids, including with longer-chain and unsaturated fatty acids [6,11,33]. 

Fatty acids CoA esters are directed to peroxisomal degradation via β-oxidation and gluconeogenesis, and key enzyme activities (such as isocitrate lyase, malate synthase and phospho*enol*pyruvate carboxykinase) have indeed been found to be specific to haustorium tissue [4,7]. Like TAG synthesis in kernel [34], the degradation of fatty acid CoA esters in haustorium is specific to medium chain fatty acids (C_10_-C_12_), longer chain fatty acids being much less efficiently (three to four times less) degraded [6]. Sugars generated by lipid remobilization and gluconeogenesis (in addition to galactomannanes hydrolysis, see above) are transiently converted to starch at stage III in the haustorium of *Butia*, macaw, coconut and oil palms [9,11,35,36]. Still, sucrose is the major product synthesized by the haustorium and exported to the developing seedling. In fact, sucrose has been found to represent up to 14% of haustorium dry weight in oil palm [11] and 5% of haustorium fresh weight in coconut palm [36].

Lipid remobilization is associated with a high requirement in CoA, since fatty acids must be esterified to CoA for metabolization. In oleaginous seeds such as canola, the CoA content increases during seed development, but declines during maturation (reviewed in [37]). Although fatty acid CoA esters are eventually cleaved and thus CoA metabolism forms a cycle (Figure 4), CoA biosynthesis is essential for lipid degradation (transport and β-oxidation) to maintain pools of intermediates and proceed at full capacity. Furthermore, in the haustorium, 0.5% only of fatty acids are present in their free form [33], suggesting that conjugation and transport as CoA esters is fast. Therefore, the de novo synthesis of CoA is an important metabolic event during germination. In *Arabidopsis*, there is a strong increase in the content of mRNA encoding for pantothenate kinase (enzyme of the CoA biosynthetic pathway) during imbibition [38]. In oil palm, the degradation of proteins, which precedes the onset of lipid remobilization is likely essential to allow CoA biosynthesis from amino acids. This includes arginine metabolism leading to β-alanine (see Non-lipid Reserves Remobilization), which is a critical intermediate in the biosynthesis of CoA. Parenthetically, it is worth noting that in plants, β-alanine comes from spermine oxidation in the peroxisome via polyamine oxidase [37], producing H_2_O_2_ and, thus, contributing to oxidative stress (further addressed below). 

Here, two important aspects are poorly, if at all, documented: (*i*) The regulation of CoA content also likely involves CoA degradation, which takes place in both peroxisomes and mitochondria in Mammals [39]. However, pathways and molecular actors of CoA degradation are virtually unknown in plants. (*ii*) Since fatty acids are transported as CoA esters, it implies that free CoA likely cycles back from the haustorium to the endosperm, once the esters have been broken down. This process can potentially take place in two ways, either via CoA transporters or CoA degradation to pantothenate that would then be transported. To our knowledge, known CoA transporters are limited to a potato mitochondrial transporter [40] and the *Arabidopsis* NAD peroxisomal transporter PNX, which can also transport CoA as well as dephospho-CoA [41]. Further research on this aspect is clearly needed to clarify how CoA homeostasis is achieved in germinating oil palm seeds and, in particular, to understand how CoA is compartmentalized between tissues (haustorium, endosperm).

## 5. ROS Metabolism

During oleaginous seed germination, there is a considerable generation of reactive oxygen species (ROS) by fatty acid oxidation. The synthesis of enzymes involved in mitigating oxidative stress has been demonstrated with proteomics in germinating *Arabidopsis* seeds [42]. In fact, acyl-CoA oxidation utilizes O_2_ either via FAD as a cofactor (via FAD-dependent acyl-CoA dehydrogenases) or directly (with acyl-CoA oxidases), thereby generating H_2_O_2_ which is then scavenged by peroxisomal catalase [31]. At this stage, it is important to remember that one H_2_O_2_ molecule is produced for each acetyl-CoA generated by β-oxidation, meaning that ROS production by β-oxidation is substantial. An increase in H_2_O_2_ concentration has indeed been found in endosperm and haustorium of germinating *Butia* palm seeds [35] and, after imbibition, superoxide dismutase and glutathione reductase activities increase, followed by catalase, thereby down-regulating oxidative stress [43]. Mitochondria are also a likely source of ROS in the first steps of germination. In seeds other than oil palm (maize, *Arabidopsis*), there are considerable changes in mitochondria shape and fusion-fission dynamics just after imbibition, with a large tubuloreticular shape comprising less cristae and a high sensitivity to KCN [44,45]. This suggests there is a low capacity of both alternative oxidase and ATP synthase, which might in turn favor mitochondrial ROS generation. Polyamines, which have also been found to facilitate oil palm embryogenesis in vitro [46], may play a role in the mitigation of mitochondrial ROS generation (reviewed in [47]). 

More generally, oxidative stress down-regulation involves the key metabolites ascorbate, glutathione and α-tocopherol. Presumably, in the case of oil palm, the relatively high amount of S-containing amino acids in reserve proteins [18] must be beneficial to glutathione synthesis, while galactomannans remobilization is a potential source of ascorbate (Figure 4). α-tocopherol is also synthesized de novo during germination, as shown in dwarf palm (*Chamaerops humilis*); in addition, in this species, α-tocopherol synthesis appears to be stimulated by H_2_O_2_, jasmonic acid (JA) and gibberellins [48]. α-tocopherol is essential to quench, not only ROS, but also lipid peroxidation triggered by lipoxygenase, which is, in turn, the source of JA [49]. In macaw palm, ROS are also generated during artificial seed ageing under wet heat which favors lipid peroxidation [50]. As such, α-tocopherol is probably an important actor to down-regulate lipid peroxidation at the very first steps of palm seed germination, just after imbibition. In fact, across different palm species, just after imbibition, there is a peak in H_2_O_2_ that seems to coincide with that in JA and α-tocopherol content [35,51,52].

## 6. Control of Germination

The means to improve germination rates have been a hot topic of oil palm biology for decades. Oil palm seeds have a mixed physical-physiological dormancy, which is often found in Monocots forming an embryo with a low degree of development in mature seeds [53,54]. In practice, it means that there is: (*i*) a physical barrier for embryonic structures to pierce the micropylar endosperm region; and (*ii*) a physiological barrier, governed by hormonal signals that need to be removed to allow germination. Importantly, the physical and physiological barriers do interact. That is, methods that tend to alleviate the physical barrier have an effect on hormones and metabolites, thereby favoring physiological dormancy breaking. Many methods have been tried to promote seed germination, such as wet heat, chemicals (such as cyanamide) or dry heat, but dry heat (39 °C for up to 10 weeks, seeds being contained in a polyethylene bag) is by far the most utilized method including in hybrids *E. guineensis x oleifera* [55,56,57]. Optimal duration and temperature have been investigated and seem to depend on the genetic material (oil palm progeny) used [58,59]. Temperature fluctuations after the induction period under dry heat have also been reported to further promote germination [60]. Interestingly, the mechanisms by which dry heat induces germination are still not totally clear. It is possible that it triggers germination via (*i*) oxidative stress—this would agree with the promoting effect on germination of both H_2_O_2_ [12,59] and high oxygen atmosphere, depending on temperature [61]. In fact, the heat treatment has been found to cause a decrease in catalase activity and an increase in glucose 6-phosphate dehydrogenase activity [62]; and (*ii*) a change in the balance between abscissic acid and gibberellins. The latter hypothesis may explain why the gibberellin GA_3_ is often added during seed treatment to further increase the germination rate, although with a rather variable success, due to the fact that GA_3_ needs to penetrate the seed, and this seems to require the mechanical piercing of the germinative pore [63,64]. Similarly, in macaw palm, the physical inhibition of germination coming from the resistance exerted by the germinative pore is not influenced by GA_3_ but GA_3_ stimulates germination by favoring embryo growth and reconfiguring cell walls of micropylar endosperm [10,51]. Additionally, in oil palm, the heat treatment has been found to decrease the force required by the embryo to pierce the germinative pore [62].

However, GA_3_ is probably not the essential factor in the natural process of germination in oil palm. Hormonal profiling of germinating oil palm seeds has shown that several hormones vary in the first days, not only gibberellins, which only show a small increase [65]. The most visible events are a strong decrease in abscissic acid and a modest increase in auxin [52,66]. The decrease in abscissic acid (and its antagonism with gibberellins) is not surprising, since it is an essential component of dormancy breaking in mixed physical-physiological dormant seeds [53,67]. In *Arabidopsis* germination, the antagonism between gibberellins and abscissic acid relies on a complicated interaction network [68], and has been shown to involve the COP9 signalosome, which mediates the regulation of protein degradation via the proteasome [69]. 

Ethylene synthesis stimulators (hydrogen cyanamide or ethephon) have also been used to stimulate oil palm germination [70], suggesting that ethylene (and/or oxidative stress, which is another consequence of hydrogen cyanamide treatment) might be involved in dormancy breaking. In macaw palm, there is more than a two-fold decrease in ACC (ethylene precursor) upon imbibition, also suggesting that ACC consumption and therefore ethylene synthesis may accompany germination induction [51]. The increase in cytokinins is visible much later (after several weeks), and is probably related to the regulation of tissue proliferation in stages III–VI [52,66]. Parenthetically, the involvement of hormones in seed germination is quite different to that in the transition of somatic embryos development in vitro. In fact, in vitro somatic embryos generated from fast-growing callus are generally cultured with anti-auxins and cytokinins and then morphogenesis is induced by GA_3_ and NAA (naphthalene acetic acid) [71]. This means that the hormone cocktail required for artificial oil palm seeds (somatic embryos embedded into a gel-based milieu) is very different from the natural hormone profile during seed germination. 

Hormones also regulate germination and dormancy-breaking via the control of metabolism. In non-oleaginous seeds such as Sichuan pepper tree (which produces starchy seeds), gibberellins inhibit SDP1-dependent lipid remobilization and increase sugar content [72]. In *Astragalus* (proteaginous legume seed), abscissic acid and methyl-JA delay lipid remobilization [65]. In oleaginous seeds, it is also probable that gibberellins and abscissic acid regulate lipid remobilization. For example, a WRKY transcription factor that acts as negative regulator of abscissic acid signaling is crucial to up-regulate lipid remobilization during germination in sunflower [73]. A comprehensive analysis of WRKY factors in oil palm has been provided [74], and future research will likely provide more insight on differential expression during the germination of WRKY factors associated with abscissic acid and gibberellin signaling. Additionally, mitochondrial reactivation during seed imbibition has been shown to be stimulated by gibberellins and inhibited by abscissic acid in *Arabidopsis* [45]. Still, specific mechanisms involving hormones in the control of metabolism in oil palm germination remain to be elucidated. Additionally, the potential interaction with nutrients is not very well documented. During kernel maturation, there is a strong decrease in the content of several elements such as Cu, Mn, Mg and K [75]. Therefore, when germination starts, there could be a transient deficiency and, consequently, it is likely that a medium enriched in such elements (or alternatively, a hormonal milieu that favors nutrient absorption) may facilitate germination. Although kernel phosphorus (P) content has also been found to decline during maturation [75], transcriptomics analyses have shown that kernel maturation is associated with the expression of genes encoding phytate synthesis [76]. This indicates that germination implies the remobilization of phytate to liberate free phosphate and sustain metabolism (such as glycolysis and ATP synthesis). In fact, in distantly related palms (*Phoenix* and *Washingtonia*), acidic phosphatase (AP) activity has been found in the endosperm and the haustorium [77]. However, the molecular mechanisms involved in AP activation are presently unknown.

## 7. Perspectives

In this brief review, we have outlined the most important events occurring in oil palm metabolism during germination. Although key metabolic pathways (such as β-oxidation) are common to all oleaginous seeds including oil palm and model plants like *Arabidopsis*, palm seeds have peculiarities, such as the involvement of the haustorium that digests the endosperm and eventually invades the seed. This is associated with metabolic imperatives such as fatty acid transport and coenzyme A cycling. Despite the importance of these aspects, advances in germination metabolism have been very limited in oil palm since the 1980s, where labelling with ^14^C has shown the interfacing role of the haustorium. It is probable that future studies will focus on functional genomics of oil palm germination. Typically, metabolomics will have to be used to resolve the spatial and temporal profile of metabolites in the different tissues of the germinating seed (as recently done in maize, [78]). Similarly, proteomics analyses will be of importance to find key transporters, confirm the localization of enzymes involved in reserve remobilization (not only lipids), and identify proteins associated with kernel oil bodies. This knowledge will in turn be useful to determine molecular traits associated with better seed germination performance and thus to help oil palm breeding.
